# Experiences of the gender climate in clinical training – a focus group study among Swedish medical students

**DOI:** 10.1186/s12909-016-0803-1

**Published:** 2016-10-26

**Authors:** Emelie Kristoffersson, Jenny Andersson, Carita Bengs, Katarina Hamberg

**Affiliations:** 1 Department of Public Health and Clinical Medicine, Family Medicine, Umeå University, 901 87 Umeå, Sweden; 2 Umeå Centre for Gender Studies, Umeå University, 901 87 Umeå, Sweden; 3 Department of Sociology, Umeå University, 901 87 Umeå, Sweden

**Keywords:** Medical education, Clinical training, Gender equality, Attitudes, Sexism, Focus groups

## Abstract

**Background:**

Research shows that medical education is characterized by unequal conditions for women and men, but there is a lack of qualitative studies investigating the social processes that enable and maintain gender inequalities that include both male and female students. In this focus group study, we therefore explored male as well as female medical students’ experiences of the gender climate – i.e., how beliefs, values, and norms about gender were communicated – during clinical training and how the students dealt with these experiences.

**Methods:**

Focus group interviews were conducted with 24 medical students (nine men) at Umeå University, Sweden. The interviews were structured around personal experiences in clinical training where the participants perceived that gender had mattered. Data were analysed using qualitative content analysis.

**Results:**

The students described gender-stereotyped expectations, discriminatory treatment, compliments, comments, and demeaning jargon. Female students gave more personal and varied examples than the men. The students’ ways of handling their experiences were marked by efforts to fit in, for example, by adapting their appearance and partaking in the prevailing jargon. They felt dependent on supervisors and staff, and due to fear of repercussions they kept silent and avoided unpleasant situations and people rather than challenging humiliating jargon or supervisors who were behaving badly.

**Conclusions:**

Everyday communication of gender beliefs combined with students’ adaptation to stereotyped expectations and discrimination came across as fundamental features through which unequal conditions for male and female students are reproduced and maintained in the clinic. Because they are in a dependent position, it is often difficult for students to challenge problematic gender attitudes. The main responsibility for improvements, therefore, lies with medical school leadership who need to provide students and supervisors with knowledge about gendered processes, discrimination, and sexism and to organize reflection groups about the gender climate in order to improve students’ opportunities to discuss their experiences, and hopefully find ways to protest and actively demand change.

**Electronic supplementary material:**

The online version of this article (doi:10.1186/s12909-016-0803-1) contains supplementary material, which is available to authorized users.

## Background

Over the past several decades, there has been an increasing number of women attending medical schools, and in Sweden and many other countries in the West the proportion of female students is today well above 50 % [1–3]. Nevertheless, conditions during studies and clinical training still differ according to gender, and studies show that many female students report difficulties in terms of limited possibilities to receive supervision or to participate in practical training [4–6]. Whereas female students report discriminatory treatment across a variety of specialties, male students mainly report such experiences during their obstetrics and gynaecology training [6, 7]. Research also shows that many female students experience sexual harassment during their education [4–8], and the prevalence of such intimidations has remained high over time [9]. However, outright discriminations and harassments constitute only a small part of the medical students’ gendered experiences, and the process of becoming a physician includes many subtle practices of gender-related inclusion and exclusion [10, 11].

It is clear that gender-related issues have important implications for medical students’ study and working conditions [12]. Thus, the importance of gender awareness in medical education has received attention in recent years [13–16], and many medical schools have included education about gender in their curricula [17–19]. However, such gender education has often been met with scepticism and resistance, and researchers in the field attribute this resistance to attitudes and values among students as well as teachers [16, 20]. This shows that formal curricular content must be viewed in the context of what is sometimes labelled the “hidden curriculum” [21], referring to the fact that many critical determinants of medical education do not operate within the formal curriculum, but through attitudes, values, and behaviours communicated in everyday interactions and clinical encounters. These informal experiences influence students’ socialization and have a powerful impact on their professional identity formation [22].

According to gender theory, beliefs and expectations about gender differences and appropriate behaviours and competence for men and women are involved in all social interactions and there are often real social costs to challenging them [23]. Gender beliefs shape and limit people’s behaviours, doings, and actions and bias their evaluations of themselves and others. Further, gender beliefs often include hierarchal dimensions where men are seen as more competent and important than women, whereas women are seen as less competent in general, but nicer and better at communal tasks, even though these tasks are less valued [23, 24].

Gender beliefs are also institutionalized within the structures and customs of organizations [23, 25], including higher education and health care organisations. In many such contexts, primary identities other than gender – such as education or occupation – might have more specific implications for behaviour, but gender always operates in the background and affects people’s activities [23]. In this light, the gendered dimensions of social interactions and work atmosphere, which are referred to as the “gender climate” in this article, are important to investigate and to understand in medical education in order to ensure equal and good treatment of both male and female students. While quantitative studies in this field regularly include both men and women, most qualitative studies have only included women even though both male and female students encounter the gender climate.

Sweden is known for taking a leading position in gender equality [26]. Almost as many women as men of working age are employed (78 % and 82 % respectively), and two-thirds of these women work fulltime [27]. Day-care facilities and support for parents in terms of pregnancy care and parental leave are well developed [28]. Today, women constitute more than half of Swedish medical students and physicians [3, 29]. However, as in other countries, men and women often choose different specialties, and male-dominated specialties tend to have higher status and/or salaries, and more men are found in high positions in health care and in academia [1, 4, 29]. There are also reports about gender-based harassment and inequalities in Swedish medical schools [5]. These facts indicate that even in countries ranking high on egalitarianism it is still important to explore gendered experiences and social processes that enable and maintain, or challenge, gender inequalities.

The aim of this qualitative study was thus to explore Swedish male and female medical students’ experiences of the gender climate in clinical work, i.e., how beliefs, values, and norms about gender were communicated during clinical training and how the students reacted to and dealt with these experiences. By including both women and men, and by being conducted in a country known for its advanced status in terms of gender equality, this study is a contribution to previous literature in the field.

## Methods

### Setting

This study was conducted at Umeå University in northern Sweden. In Sweden admission to medical school takes place twice a year. The curriculum includes 5.5 years (11 terms) of education, and the last 3 years include clinical training for about half the time. The proportion of female medical students in Umeå has been 50–60 % for the last 10 years. In Sweden, no registration is made of medical students’ class background or ethnicity, but questionnaires administered to students in Umeå in 2006–2009 showed that the large majority were middle class and less than 10 % of the students reported that one or both parents were born outside of Scandinavia [13].

In 2001 the board of the Umeå University Medical School decided to include education about gender issues in the basic curriculum. This implied that in addition to including the biological, social, and cultural aspects of being a male or a female patient, gender beliefs and equality should also be part of discussions about the role and career of physicians and students – from year one onwards [12, 17].

### Design, participants, and data collection

This qualitative study consisted of focus group interviews [30] conducted in 2012 and 2013. Medical students in three senior classes (terms 9–11) with 80 students each were invited by the first author to participate via presentations after lectures and via e-mail sent to all students. Only e-mail invitations were sent to students in term 10 because this class hardly had any lectures at which to give a verbal presentation. Both in the verbal presentation and in the e-mail, the students were informed about the purpose of the study and about how to get in contact with the researchers.

All eighteen students (15 women) that volunteered were included and divided into four focus groups. Two groups consisted of only women and two were mixed. To include more men, we later recruited six male students (from term 8–11) by ‘snowball sampling’ into a fifth group. In total, 24 students (15 women and 9 men), all in their mid-twenties to mid-thirties, participated. The purpose of having two mixed groups was to give the students direct access to experiences that they perhaps otherwise might not hear about.

To facilitate deeper insights and reflection, groups one to four were interviewed twice with about one month between the interviews. Two women did not participate the second time. Only one interview was conducted with group five. The first and second authors, EK and JA, conducted five interviews together alternating between the position of moderator and observer, and EK conducted three follow-up interviews on her own. In the fifth all-male focus group, a male research assistant took part in the data collection as the observer together with EK as the moderator. The interviews lasted 50–95 min, were recorded digitally, and were transcribed verbatim.

Each interview started with a short discussion about the importance of confidentiality of what was disclosed in the focus group. The students were then asked to share their own experiences of situations in clinical training where they perceived that gender aspects had mattered. They were also asked about their own reactions and ways of acting and dealing with the described situations. Follow-up questions like “Can you give an example?” or “What do you think when you hear this narrative?” were posed to develop the answers and to invite other participants into the discussion. (The interview protocol can be found in the Additional file 1). After each interview, EK and JA listened to the recording to identify aspects that needed elaboration in the follow-up interview with the same group, or in interviews with other groups. In the follow-up interviews, the moderator began by briefing the participants with a short summary of what came up in the previous interview, and then gave the participants time to reflect, comment, and give further examples before any new questions were posed. When the interview with the fifth all-male-group was conducted and listened through, we found that the new examples and experiences that came up were of the same character as in the previous interviews and we decided to stop the data collection.

In this article, detailed background information for participants has been withheld, and details about staff occurring in the students’ descriptions have been deleted or changed to ensure confidentiality.

### Analysis

According to qualitative research design [31], the analysis started in parallel with the data collection by listening to the recordings. This procedure made it possible to correct potential misunderstandings and get complementary examples that could contribute to variation and to nuanced interpretations.

The main analysis was inspired by qualitative content analysis [32]. Initially, EK and KH (last author) read all interviews to get an overview of the material. The text was then explored for meaning units consisting of utterances or sentences of relevance for the study aim. These meaning units were condensed and shortened, but with a retained core message. The condensed meaning units were later assigned codes and sorted into domains, i.e. areas of questioning. Codes in the same domain were compared for similarities and differences regarding meaning and content to generate subthemes and themes. Codes and related meaning units in the same subthemes were also compared for similarities and differences between male and female students. EK and KH separately explored and coded the text, and they met regularly to discuss their interpretations and to develop the analysis. In order to enhance the trustworthiness of the analysis, the other authors also analysed two interviews and then all authors met to discuss and further elaborate on domains, subthemes, and themes [33]. Table 1 gives an example of the steps of the analysis.

**Table 1:** Illustration of the coding procedure from condensed meaning units to themes

Condensed meaning units	Codes	Sub-themes	Themes
To win confidence, I act like “one of the guys” or use my charm	Acting like one of the guys Using my charm	Taking part in the jargon	Manoeuvring to manage
I try to be ugly to avoid harassment	Adapting appearance	Adapting themselves	
Weird atmosphere when I kept distant	Kept away	Avoiding	
My supervisor said to the patient: “I have a young, pretty girl here, it’s okay if she tags along, right”?	Comments on appearance	Compliments and comments	Facing compliments, comments, and jargon
The head physician continuously made derogatory jokes about women	Doctors making derogatory jokes about women	Tough jargon	
I had to give him a certain kind of attention	Demands for a certain attention	Sexual innuendos and invitations	

## Results

The interviews seemed to act as a catalyst for reflection and recollection of personal experiences, and the material was rich in examples. Many participants initially stated that gender did not matter, but they later described numerous situations where they had noticed that gender aspects were important. The heart of the students’ discussions was on negative or problematic experiences. A recurring dimension in female as well as male students’ descriptions was the experience of being dependent on supervisors or staff to obtain good clinical training and to learn what they were supposed to learn. This dependent position contributed to feelings of vulnerability and affected their reasoning and acting. Overall, the women described more concrete situations and gave more varied examples where they were personally involved than the men did. Our analysis is presented in two domains: ‘Experiencing gendered conditions’ and ‘Navigating from a dependent position’. Both domains consist of a number of themes and subthemes (see Table 2).

**Table 2:** Summary of the analysis in domains, themes and sub-themes

Domains	Themes	Sub-themes
Experiencing gendered conditions	Meeting stereotyped expectations	Traits and characteristics
		Family and career
		Proper behaviour
		Care and emotional support
	Encountering discriminatory treatment	Not getting the chance to participate
		Not being taken seriously
		Being made invisible
		Favouring of men
	Facing compliments, comments, and jargon	Compliments and comments
		Tough jargon
		Sexual innuendos and invitations
Navigating from a dependent position	Individual processing	Blaming themselves
		Diminishing
		Redefining
		Blaming others
	Manoeuvring to manage	Adapting themselves
		Taking part in the jargon
		Avoiding
	Acting for change	Talking back and protesting
		Seeking/giving support

In the presentation of the domains below, the themes are given as headlines in italics and the subthemes are marked in italics within the text. Quotations from the students are interspersed to ground the presentation in the students’ accounts.

### Domain one: experiencing gendered conditions

#### Meeting stereotyped expectations

The students depicted a clinical environment where women and men were met with different expectations regarding their *traits and characteristics* and their ambitions for *family and career*. Female students were expected to be conscientious, hard working and responsible, but were also assumed to be insecure and to put a lot of pressure on themselves. They felt that it was often presupposed that they would take primary responsibility for family and children and they were, therefore, warned against certain specialties. Occasionally, supervisors referred to physical strength to explain opinions about unsuitable specialties for women:

“A female classmate was told that it wasn’t appropriate for her to become an orthopaedist because it was too hard physically, it was more of a man’s job.” (Male student, M)

Men, on the other hand, were expected to be self-confident and forward, and they were seldom asked about family plans or advised about specialties to avoid because they were men.

According to the students, norms about *proper behaviour* were also gendered. Whereas men were mainly evaluated based on their skills and clinical performance, women had other aspects of their behaviour scrutinized as well, for example, their way of dressing or using makeup. They therefore often felt restricted:

“The scope of action for what women can do and what is considered okay is so much narrower than it is for men.” (Female student, F)

Gender also had implications for what students were expected to do in terms of *care and emotional support* at the clinics. Most female students had been called “nurse” by patients or staff, despite presenting themselves as medical students. Most female students, but no male students, had also been asked by staff or patients to do caring tasks, like helping patients put on their shoes or to go to the toilet. Some women felt that they, as women, were per se seen as emotionally competent and were therefore expected to take care of and give emotional support to sad patients. A couple of the female students described situations where they had been caught of guard by a hug from a patient, which they felt was because they were seen as too emotionally available.

### Encountering discriminatory treatment

Several female students had encountered unfair treatment during their clinical placements by *not getting the chance to participate* in certain training or not being offered the same opportunities for supervision as their male peers. Others talked about *not being taken seriously* or of *being made invisible*, like in this example:

“The doctors never wanted to acknowledge my presence. Even when I asked questions, the answers were directed to the male students.” (F)

Several male students had also noted the unfair treatment of female peers, and they also reported own experiences of discriminatory treatment. These mostly took place during their placements in gynaecology and obstetrics, and some had not been allowed to participate or observe intimate examinations or deliveries. In most clinics, though, the situation was the opposite with a subtle *favouring of men* when distributing tasks, giving access to medical interventions, or offering support and supervision. This male participant reflected:

“At the ward, a nurse offered to find a suitable patient for me, showed me a room to work in, and gave me the patient’s social security number… Later a female peer came and I noticed a very big difference in the reception she got; she wasn’t offered the same help.” (M)

Female students seemed to expect a certain understanding and solidarity from female physicians. One student felt confirmed and relieved when a female supervisor emphasized that achieving authority, as a physician might be harder for women:

“Our female supervisor said, ‘You girls have to work extra hard because it is more difficult for you to be taken seriously.’” (F)

When discussing this example in the group, some other women meant that even if the instructor’s intentions might have been good, this was just another annoying example of discrimination showing that women have to manage greater challenges than men.

### Facing compliments, comments, and jargon

Several female students had received *compliments and comments* from male supervisors and staff about their own or others’ appearance. Some were derisive comments about women who were regarded as unattractive, but most comments could be characterized as compliments. Both types were described as problematic, and the female students wanted to receive attention for their competence, not their appearance:

“To me it feels like it affects my role as a doctor-to-be negatively if someone says I’m pretty.” (F)

Some men had received compliments from female patients or nurses, but none of them identified it as a problem. This male participant even depicted it as a positive achievement:

“Well, I’ve managed to charm a couple of old ladies in my days, many of whom have said that I’m cute.” (M)

The students also described how a *tough jargon* dominated some clinics and how this jargon was characterized by rude comments, epithets like “honey” or “good girl”, and recurrent jokes with a derogatory or sexual content. Women were depicted as the main targets for the comments and mockery. Coffee rooms and operating rooms seemed to be typical contexts for such jargon:

“The male doctors talked about their favourites among the female interns, and the chief physician said, ‘Helena was more intelligent, but Anna was prettier so she was my favourite.’” (F)

Several female students had experienced flirting and *sexual innuendos and invitations* from supervisors, staff, or patients. Some had, for example, been invited for a date by male supervisors. For one of these women the situation became uncomfortable and hostile when she turned the supervisor down. Other examples concerned male patients who gave hints about sexual interest.

One male participant described an incident of being inappropriately touched by a female nurse, but overall the men had few experiences of being the subject of jokes, sexual innuendos, or advances.

### Domain two: navigating from a dependent position

#### Individual processing

The students described their experiences of the gender climate and how beliefs and attitudes toward men and women came to the surface in the clinic as surprising or even shocking, and feeling belittled or humiliated was a common reaction.

The women described how demands for providing care and emotional support gave rise to stress and irritation because such work was ambiguous and time-consuming. Flirtation and compliments aroused conflicting emotions; they felt flattered but at the same time ashamed over getting appreciation for their looks instead of for their work. Some said it was easier to handle comments and sexual innuendos from patients than from staff because patients were beneath them in the clinical hierarchy. They also excused patients’ behaviour by their being ill or old.

Although being irritated and angry, the women often *blamed themselves* when they were subjected to unfair treatment or they *diminished or redefined* such incidents as a matter of individual interpretations and thereby renounced their own reactions. Some blamed their young age for the treatment they received. Several women were reluctant to share their stories with others at the clinic because their reactions had previously been questioned:

“If I tell a male classmate that I was patted on the head or that I was called ‘honey’, I’m often met with comments such as ‘There was nothing to that!’ or ‘It’s not because you’re a woman, it hasn’t got anything to do with that.’” (F)

The male students who had experienced bad or discriminatory treatment were also frustrated and sometimes angry. This student was disappointed when he was rejected from a gynaecological examination:

“They judged me based on being a man, not on my personal characteristics.” (M)

The male students were often *blaming others* when they felt neglected or mistreated. Some who had not been allowed to participate during deliveries in the maternity ward described how the staff contributed to their difficulties by reinforcing patients’ doubts about male students or by being negative themselves to men’s participation:

“The midwife just looked at me and said, ‘You can watch childbirths on film.’” (M)

Other male students stated that as men and physicians-to-be they were in a position of power in relation to patients and nurses, and that this in some sense protected them from bad treatment.

#### Manoeuvring to manage

In various ways, the students tried to manoeuvre and prevent negative situations, maintain smooth working relationships, and meet perceived expectations by *adapting themselves*. As a way to handle not feeling welcome in gynaecological examinations, some men had tried to be extra considerate and gentle. To evade harassment and comments about being cute or pretty and to prevent people from believing that they tried to make use of their femininity, a recurring manoeuvre among female students was to adapt their appearance by wearing less makeup and keeping their hairstyle conservative. Female students also described how they tried to make good connections and to adapt and fit in with the nurses through discussions about hobbies and family life.

When overhearing derogatory jokes from instructors or supervisors, both male and female participants felt expected to play along and laugh, which they often did. They also described that *taking part in the jargon* and flirting or playing on gender stereotypes could be a way to create better circumstances for themselves. Some male students said that by flirting with female nurses they could get benefits:

“You have a huge advantage being a guy. The nurses are a lot more receptive, helpful, and attentive towards you. And I don’t think one can be blamed for making use of it. Being a little flirty, groovy, and seductive, you can get a lot out of it.” (M)

Several women disclosed how they had given affirmation or attention to male supervisors with the secret motive of gaining supervision or escaping repercussions. They often outlined their use of flirting and flattery as ambiguous and somewhat shameful, and they reflected on their own role in partaking in the reproduction of a gendered atmosphere:

“I don’t want it to be my lifeline to flatter and play games, but with well-tried experience it’s what has worked. Sometimes I’m disgusted with myself even though it’s due to self-preservation. I’m not proud of it anyway.” (F)

Being careful and *avoiding* uncomfortable situations was a common strategy for dealing with a problematic working atmosphere and unfriendly comments from supervisors or staff.

“To avoid the sexual innuendos I kept myself a bit distant.” (F)

However, keeping away from unpleasant people and potentially uncomfortable or nasty situations implied a risk for being marginalized and missing important training.

#### Acting for change

Taking action when being exposed to or witnessing bad treatment, such as *talking back and protesting*, was described as empowering by the participants. Few concrete examples of this were given, however. Some students – both men and women – said that showing disapproval or talking back was pointless because no one would listen or that protests might create new problems. This student reacted strongly towards a supervisor who uttered homophobic, racist, and misogynist opinions, but because he needed help from this supervisor he found it difficult to act:

“I needed this person’s help for the whole week so I couldn’t afford to have any conflicts with him. I would have suffered from doing so.” (M)

Others described how showing irritation, or just being silent, carried risks of negative consequences, like this woman:

“The surgeon told a sexual joke, but I didn’t laugh. Instead I said, ‘Felt that was funny did you?’… After that, I did not know where to go because the atmosphere became so tense.” (F)

*Seeking support* and confirmation from others was an important strategy used by several students to manage and process their experiences, as well as daring to act. This student turned to her classmates for support when being neglected and badly treated by a supervisor, and this provided her with confirmation that she was not just imagining things:

“It felt good when I brought it up. Another student in my group, a guy, had also tried to point out the problem to the supervisor. So it wasn’t just all in my head. I felt that they understood.” (F)

This quotation also illustrates that male as well as female bystanders to harassments and bad treatment could play an important role by *giving support* to the person who was being harassed.

## Discussion

This study gives insights into medical students’ experiences of the gender climate and how beliefs, values, and norms about gender were communicated during their clinical training. The students described being met with stereotyped expectations about men and women, discriminatory treatment, compliments, comments, and rude or demeaning jargon. The women described many situations where they were personally involved, while the men mainly talked about incidents they had heard about or had observed as bystanders. Common ways to manage their experiences were to diminish or redefine what had happened, to adapt their appearance, to partake in jargon, or to avoid unpleasant situations, people, and places. The participants emphasized the importance of taking action for change but found this to be difficult. Male and female students shared the experience of being dependent on supervisors and staff to obtain good clinical training and this characterized their stories as well as their reasoning and their behaviour.

### Future physicians facing old stereotypes

All participants wanted to be seen as physicians-to-be rather than as men and women. Still, expectations about gender differences, including appropriate behaviours for men and women as well as hierarchies concerning gender, were crucial dimensions in their narratives about their interactions with supervisors, staff, and patients. The medical profession and its associated traits, abilities, and activities, is historically and culturally linked to men [34], and in such contexts beliefs about men having higher competence and being more credible than women become extra prominent [23]. In accordance with research on medical education from other countries [11, 16], our results showed that such stereotyped presumptions about men and women seem to bias teachers’ and physicians’ expectations and evaluations of students’ performance.

In line with previous studies, our male students reported their own experiences of mistreatment, mostly during placements in gynaecology and obstetrics [6, 7]. With that exception in mind, both male and female students described a subtle favouring of men throughout clinical training. Corresponding to ideas of men’s competence and skills [23, 24, 35], male students were taken more seriously as physicians-to-be, were seen as confident, and were provided with a greater scope of action than their female peers. Further, being men and positioned higher in the gender hierarchy might also explain why they saw compliments and comments from female patients and nurses as unproblematic. The male students could also flirt with only minor risks of social sanctions, and they sometimes engaged in such behaviour to further improve their position among female staff.

Much of what emerged in the interviews about the gender climate and difficulties for the female students in particular could be described as ‘everyday sexism’, referring to incidents and interactions that individually might seem trivial, but where the cumulative effect of each pejorative joke, prejudiced assumption, and degradation of women helps to uphold unequal social relations [11]. Sexism is usually associated with hostile prejudices, implying that women are less worthy of status and power than men [24], and this was seen in the interviews as being disregarded and made invisible and through derogatory jokes about women. According to a previous Swedish survey, the most common forms of gender-related discrimination reported by female undergraduate medical students is to be ignored or not taken seriously [5]. Our female students’ fear of being accused of making use of their femininity to achieve benefits is also a mirror of hostile sexist ideas implying that women deliberately use sexuality to gain power from men [24].

However, sexism can also take affectionate or seemingly benevolent expressions, rooted in ideas that women have unique superior qualities, e.g., that they are particularly patient, meticulous, sensitive, and fragile and therefore in need of admiration and protection – and are best suited for the conventional roles of women [24, 36]. The female students’ experiences of warnings against unsuitable and physically demanding specialties, and compliments about their appearance, reflect such prejudices. That sexism can be expressed not only through hostile comments but also through paternalistic protection was also recently shown in a study among medical students in Taiwan [37]. The fact that the women in our study were viewed as insecure but responsible and hard working meant that their achievements risked being seen more as an outcome of diligent efforts than being the result of their intelligence and aptitude for medical work. In line with ideas of women’s talent for communal tasks [24], our female participants were expected to provide care and emotional support and were presumed to prioritize family in a way that would risk their careers. In that light, their frustration about demands on care and emotional support, as well as being mistaken for nurses, becomes even more understandable because, in addition to a heavier workload, such incidents pinpointed that they risk being devalued in the health care organisation.

The female students had received compliment and comments from male supervisors who were above them in the clinical hierarchy. The women’s hesitance to protest against the seemingly positive attention is thus understandable. So to escape these comments, they kept distant or adapted their appearance. What made the female students act flirtatiously towards male supervisors was sometimes a desire for better conditions for themselves – but every so often it was because of a fear of punishment if they did not respond positively to a supervisor’s compliment or joke.

### Reasons for ‘not seeing’, and incentives for passivity

Many participants initially stated that gender did not matter, but they later described numerous experiences of constraining gendered preconceptions, discriminatory treatment, and demeaning jargon. Why did the students have difficulties in recognizing or acknowledging gender-related mistreatment? One explanation might be that being the victim of discrimination is in conflict with political and social norms about Sweden being a gender- equal country, as well as with contemporary liberal ideas of individual independence and responsibility [38]. To face discrimination was therefore surprising and induced feelings of shame and personal failure, leading the female students to often blame themselves for causing the unfair treatment instead of actively protesting such treatment. A second explanation is related to the strong ideals in medicine about objectivity and neutrality that have been shown to socialize medical students to believe that aspects like gender, as well as class, ethnicity, and sexual orientation, are, or should be, insignificant for their own working conditions [20, 39, 40]. Students’ adaptation to such ideals might explain why some of the female students had their experiences of unfair treatment questioned by their peers. Thirdly, it is hard to identify and communicate how benevolent sexism like positive comments and compliments can in fact be a way to downgrade and diminish a person [24, 36]. These three mechanisms might all contribute to students’ being blind to or not wanting to see gender-related mistreatment.

Furthermore, when recognizing gender-related discrimination or bad treatment the students seldom acted against it. They described themselves as being dependent on supervisors and staff, and their quest to become accepted in the clinic and to receive guidance created powerful incentives to not challenge norms, or to stay silent when facing or overhearing abuse or negative treatment. However, this silence means that the individual disadvantaged students, on most occasions women, are left alone to find strategies to handle bad treatment unless they seek support or are offered support from others. That today’s students are trying to adapt to, avoid, and sustain gender prejudices, instead of taking action for change, indicates that future physicians risk redefining structural problems into individual shortcomings and that they risk reproducing a negative gender climate in the clinic.

### Methodological considerations

We put out a broad invitation to recruit medical students to this focus group study, but it was still difficult to recruit men. Previous studies have identified a lack of interest and lower gender awareness among male medical students [13, 17], and this might explain our recruitment difficulties. Due to confidentiality and the fact that most students in Umeå come from white middle class families, we left considerations regarding ethnicity, class, and sexual orientation out of the analyses. The procedure with follow-up interviews enabled us to get complementary examples from some of the students who had reflected after the first interview and had more examples they wanted to share.

A potential weakness of the study design could be that some focus groups included both men and women. For example, female students in the mixed groups might have been afraid of disclosing gendered experiences if they were worried that male students in the group would be critical or try to dismiss their experiences. However, the character of the discussions and the male and female students’ examples in the single-sex groups were similar to the discussions and examples occurring in the mixed groups. As a result, each sub-theme was derived from a number of descriptions and examples and related to accounts made by students in both mixed and single-sex groups.

Nevertheless, in focus group interviews, hierarchies and norms always affect what participants choose to say [30]. This could mean that the students adapted their narratives to what they thought the interviewers and other participants wanted to hear. However, the fact that the students described sensitive situations when they had felt exposed, downgraded, and marginalized – as well as their own partaking in gendered jargon and other behaviours they were ashamed of – indicate that the discussion climate was rather open.

Although Sweden gets high scores when gender equality is measured [26], our students painted a picture with many examples of gender stereotyping and mistreatment, a picture appearing surprisingly similar to descriptions from students in countries with lower gender equality scores [9]. This was an important result but also a condition that creates questions about how to compare our findings with studies from other contexts. It might be that Swedish students, or students in the Umeå University Medical School, are more gender aware and thus are more likely to notice gender-related problems, or react to more subtle problems, than students in less gender-equal cultures and medical schools. Further, the students who volunteered for this study were probably more interested in gender issues and more used to discussing and scrutinizing gender inequality than Swedish students on average. Still, we have no reason to doubt our students’ narratives and we believe that the character of their experiences, considerations, and ways of coping are transferable to other medical students in Sweden and many other Western countries.

## Conclusions

The widespread, everyday communication of gender beliefs combined with the students’ adaptation to sexism and gender discrimination came across as fundamental features through which unequal conditions for male and female students were reproduced and maintained in the clinic. Consequently, gender remains an important aspect in the process of becoming a physician.

To reduce gender stereotyping and biased treatment and to obtain equal conditions for male and female students, it is important for medical schools to include gender education in the formal curriculum but also to create opportunities for teachers and staff to develop their gender competence. Medical schools need to provide students, teachers, and supervisors with theoretical concepts and knowledge to help them understand gendered processes and recognize discrimination. Because compliments are often seen as purely positive, supervisors and students need to learn about and discuss the effect of not only hostile, but also of benevolent sexism. To arrange groups for reflection about the education climate and everyday sexism in the clinic is suggested to be an important intervention for improving both students’ and supervisors’ abilities to recognize and to act against gendered mistreatment.

Undesirable attitudes and gender discrimination are hard to challenge by single individuals, especially if they are in subordinate positions like students. Therefore, the main responsibility for creating a study and working climate where openness, protests, and discussions about changes of attitudes are possible lies with the leaderships of medical education and health care organisations. We have no reason to believe that changing the gender climate will be a smooth and easy-going process, and we therefore suggest that heads of medical education should allocate economic resources and experts to this change process and to especially welcome and include suggestions from students.

## Additional file

Additional file 1: This file contains the interview protocol used in the focus group interviews. (DOCX 110 kb)
