# Supplementary material for: Experiences of the gender climate in clinical training – a focus group study among Swedish medical students
Source: BMC Med Educ. 2016 Oct 26;16:283. doi: 10.1186/s12909-016-0803-1 (PMC5082355; doi:10.1186/s12909-016-0803-1)
Supplement: Additional file 1: — This file contains the interview protocol used in the focus group interviews. (DOCX 110 kb) [file 12909_2016_803_MOESM1_ESM.docx]

Additional file 1

**Interview protocol**

A. General information to the participants as a start

Welcome. The moderator repeats the previously given information about the background and aim of the study, the structure and time frame for the focus group interview, the importance of confidentiality within the group, that the data will be used for research purposes, that in the published results the participants will not be recognizable, and that participation is voluntarily and a participant can stop her/his participation at any time during the process.

Participants are given the opportunity to ask questions before the interview begins.

B. Interview questions

Can you say a little about your experiences of being a medical student in clinical training?

How are students treated at the clinics? Can you describe a good/bad clinical placement? Why was it good/bad?

What is your overall impression about the importance of gender issues in clinical work? (Follow up questions: In what way did gender matter? Can you give an example?)

Can you describe a situation where gender mattered in a problematic or negative way? (Follow up questions: Can you explain more about it? Who did what? How did you feel/react? How did you deal with the situation? What do you think when you hear this narrative?)

Can you describe a situation where gender mattered in a positive way – or when a gender issue was handled in a good way? (Follow up questions: Can you explain more about it? Who did what? How did you feel/react? How did you deal with the situation? What do you think when you hear this narrative?)

Can you describe a situation that you have experienced, observed, or heard about where you or any of your classmates have experienced any unfair treatment or discrimination? (Follow up questions: How did you feel/react? How did you deal with the situation? What do you think when you hear this narrative?)

Does physical appearance matter at the clinic? In what way? Can you give an example?

Have your (gender related) experiences from the clinic affected how you think about your future career? In what way? (*Not included in this manuscript*)

Do you have any good/bad physician role models? Can you describe them? (*Not included in this manuscript*)

Is there anything that you would like to add before we end the interview?
